# “Assessment of diabetes management strategies for blood glucose control in Sana’a City General Hospitals”

**DOI:** 10.3389/fendo.2025.1587901

**Published:** 2025-07-17

**Authors:** Ali Salman Al-Shami, Mokhtar Alzumor, Adnan Aladhal, Yaser Al-Worafi, Zaid A. Thawaba

**Affiliations:** ^1^ Department of Pharmacology, Faculty of Medicine, Sanaa University, Sanaa, Yemen; ^2^ Department of Pharmacy, Jiblah University for Medical and Health Science, Ibb, Yemen; ^3^ Department of Laboratory, Faculty of Medicine and Health Sciences, AL Nasser University, Sanaa, Yemen; ^4^ College of Medical Sciences, Azal University for Human Development, Sanaa, Yemen

**Keywords:** diabetes mellitus, blood glucose control, management strategies, laboratory monitoring, Yemenis patients, blood bio-markers

## Abstract

**Background:**

Diabetes mellitus is a major global health concern associated with serious complications. Effective management of blood glucose levels is crucial in reducing these complications.

**Objectives:**

This study aimed to evaluate blood glucose management strategies among diabetic patients in general hospitals in Sana’a city, Yemen.

**Methods:**

A cross-sectional study was conducted involving 50 diabetic patients. Data on demographics, risk factors, treatment adherence, lifestyle habits, and biochemical markers were collected through questionnaires and laboratory tests. Statistical analysis was performed using SPSS.

**Results:**

The study found a higher prevalence of diabetes among males, individuals aged 41–60 years, married persons, and those who were illiterate. “Type 2 Diabetes Mellitus was more common than insulin-dependent diabetes mellitus (IDDM) (80% vs. 20%). Laboratory monitoring was the most commonly used management strategy, while adherence to treatment, exercise, and nutrition was lower. Patients adhering to treatment, exercise, and nutrition therapy showed significantly better clinical and biochemical profiles, including lower LDL, triglycerides, total cholesterol, HbA1c, fasting blood sugar, BMI, and blood pressure levels. The prevalence of diabetes complications was lower among patients who followed management protocols.

**Conclusions:**

Regular treatment adherence, exercise, nutrition therapy, and laboratory monitoring contribute to improved blood glucose control and reduce diabetes-related complications. Enhancing patient education and adherence to management strategies is essential for better clinical outcomes.

## Introduction

Diabetes mellitus (DM) is a very common metabolic disease characterized by high glucose levels in the blood due to dysfunction in the pancreas to produce sufficient amounts of insulin hormone. Diabetes Mellitus (DM) is likely one of the oldest diseases recognized by humanity. The earliest documentation of this condition can be traced back to an Egyptian manuscript dating approximately 3,000 years ago (1) Treatment of almost all types of DM became available when insulin was discovered and produced in 1921 (2). Management of type 2 diabetes mellitus can be achieved through lifestyle modifications, dietary changes, and the implementation of available pharmacological treatments (3). Diabetes mellitus significantly contributes to morbidity, ranking as one of the leading causes of renal failure, blindness, and limb amputation in adults. Additionally, it plays an often-underestimated role in overall mortality (4). The origin and etiology of diabetes mellitus can vary significantly, but they consistently involve defects in either insulin secretion or insulin resistance at some stage of disease progression. Patients with diabetes mellitus typically present with either type 1 diabetes immune mediated or idiopathic or type 2 diabetes mellitus, previously referred to as non-insulin-dependent diabetes mellitus. Type 2 diabetes is the most prevalent form of the condition, characterized by hyperglycaemia, insulin resistance, and relative insulin deficiency (ADA) (3) Research indicates that rigorous metabolic management may postpone or prevent the development of complications associated with diabetes (5). The results of large randomized trials involving individuals with type 1 diabetes or newly diagnosed or established type 2 diabetes demonstrate that effective glycaemic control can delay the onset and slow the progression of microvascular complications, such as nephropathy, retinopathy, and neuropathy (6). The needs of diabetic patients extend beyond achieving adequate glycaemic control. Effective management also includes preventing complications, limiting disability, and facilitating rehabilitation. Maintaining blood glucose levels within the target range is a primary goal in the treatment of diabetes mellitus. Data from the Diabetes Control and Complications Trial (DCCT Research Group) show that in individuals with type 1 diabetes, a 10% reduction in glycated hemoglobin (GHb) levels was associated with a 43% decrease in the risk of retinopathy progression (7). Findings from the Gallichan study, further indicated that morbidity associated with diabetes mellitus—as well as the use of healthcare resources for managing diabetic complications—could potentially be reduced by significantly improving blood glucose control (8). Although various methods exist for blood glucose monitoring, the associated costs can be substantial. Gallichan estimated that the annual cost of self-monitoring by diabetes mellitus patients within the National Health Service (NHS) was approximately £42 million (8) Factors to consider when establishing glycaemic goals include psychosocial limitations (9). In patients with hypoglycaemia unawareness, glycaemic targets should be adjusted to less stringent levels over extended periods, with the aim of potentially reversing the condition (10). For patients with severe comorbidities that may hinder management strategies, the objective shifts to preventing clinically significant glycosuria, fluid and electrolyte loss, infections, and the onset of non-ketotic hyperosmolar coma. Insulin is prescribed for individuals with type 1 diabetes and for those with type 2 diabetes who exhibit insulinopenia and do not respond adequately to dietary therapy or oral hypoglycaemic agents (11). In such cases, insulin therapy may be the most effective method for reducing blood glucose levels (12). A significant proportion of patients with type 2 diabetes will ultimately require insulin therapy. Due to insulin resistance, these patients may need doses exceeding 1 unit per kilogram of body weight per day (13). Recent studies suggest that hyperglycaemia in hospital settings is not a benign condition. Intensive treatment of diabetes and hyperglycaemia has been associated with reduced morbidity and mortality. Evaluations of inpatient hyperglycaemia management have emphasized the importance of glycaemic control in improving hospital outcomes (14) The overarching goal of diabetes management is to maintain blood glucose levels near normal while avoiding hypoglycaemia, with an HbA1c target typically between 7% and 8% (15). This is generally achieved through a combination of dietary modification, physical activity, weight loss, and appropriate use of medications, including insulin and oral agents. Complications occur less frequently and are less severe in individuals who maintain good glycaemic control. Type 1 diabetes must be managed with insulin injections. In contrast, the prevention and treatment of type 2 diabetes involve following a healthy diet, regular physical activity, maintaining a normal body weight, and avoiding tobacco use. Pharmacological treatment may include insulin sensitizers, with or without insulin (16). Additionally, managing blood pressure and ensuring proper foot and eye care are critical components of diabetes management (17).

## Methodology

### Study design

The investigation utilized a cross-sectional study design. Conducted in several general hospitals, including Al-Kuwait, Alsabeen, Republic, and 48, other hospitals located in Sana’a city, Yemen. The study population comprised all individuals diagnosed with diabetes mellitus at the participating hospitals, with a total of 50 diabetic patients prospectively enrolled, ranging in age from 21 to 90 years.

### Inclusion criteria

This study included all patients with diabetes mellitus who consented to participate, met the inclusion criteria, and were hospitalized on the days of the student researchers’ visits.

### Exclusion criteria

The study excluded patients who refused to withdraw their samples or complete questionnaires, as well as those with hyperthyroidism, pancreatitis, Cushing’s syndrome, glucagonoma, heart attack, or who were taking glucose-raising medications such as estrogen, glucagon, prednisone, or oral contraceptives.

### Data collection

The survey included a self-interview and an administered questionnaire with the diabetic patients. The questionnaire was designed to include personal information like name, age, sex, occupation, address, and level of education, as well as information about daily habits and diets, clinical information like weight, height, and blood pressure, medical histories of other diseases, and risk factors and complications related to DM.

### Limitation

This study has several limitations that should be considered when interpreting the findings. First, the relatively small sample size of 50 diabetic patients limits the statistical power of the analysis and reduces the generalizability of the results to the wider diabetic population in Sana’a city and Yemen. Secondly, the recruitment was limited to hospitalized patients present during the researchers’ visits, which may introduce selection bias by overrepresenting patients with more severe disease or complications. This sampling method limits the applicability of findings to community-dwelling or outpatient diabetic populations who might have different management experiences and outcomes.

Additionally, the cross-sectional design provides only a snapshot of diabetes management and does not allow for assessment of causal relationships or changes over time. Self-reported data on treatment adherence, exercise, and nutrition may be subject to recall bias or social desirability bias, potentially affecting the accuracy of reported behaviors.

Finally, some biochemical and clinical parameters were measured only once, which may not fully capture fluctuations in blood glucose control or other metabolic variables.

Future studies should address these limitations by including larger, randomized, and more diverse patient populations, employing longitudinal designs, and incorporating objective measures of adherence and glycemic control.

### Statistical analysis

The data were analyzed using SPSS Version 20 (Statistical Package for the Social Sciences), checked for normal distribution, and expressed as percentages, mean ± SD. Independent sample T tests and Chi-square tests were employed to assess differences in variables, while risk factors were estimated using odds ratios (OR) through binary logistic regression. ANOVA was used to look at the important connections between the parameters. Differences were considered significant when the P-value was less than 0.05.

## Results

**Table 1** indicates that a significant proportion of patients were married (94%), unemployed (56%), and illiterate (40%). The age group most affected was 41–60 years, comprising 52% of cases, with a slight predominance observed among males at 52%. These factors indicate a possible association between socioeconomic vulnerability and diabetes prevalence.

**Table 1:** The sociodemographic characteristics of the studied diabetic patients.

The sociodemographic characteristics		N	%
Social Status	Married	47	94.0
	Single	2	4.0
	Divorce	1	2.0
Educational Level	Illiterate	20	40.0
	Primary	4	8.0
	Preparatory	6	12.0
	Secondary	9	18.0
	Diploma	1	2.0
	Bachelor	10	20.0
Occupation	Employee	22	44.0
	Non-employee	28	56.0

**Figure 1:**
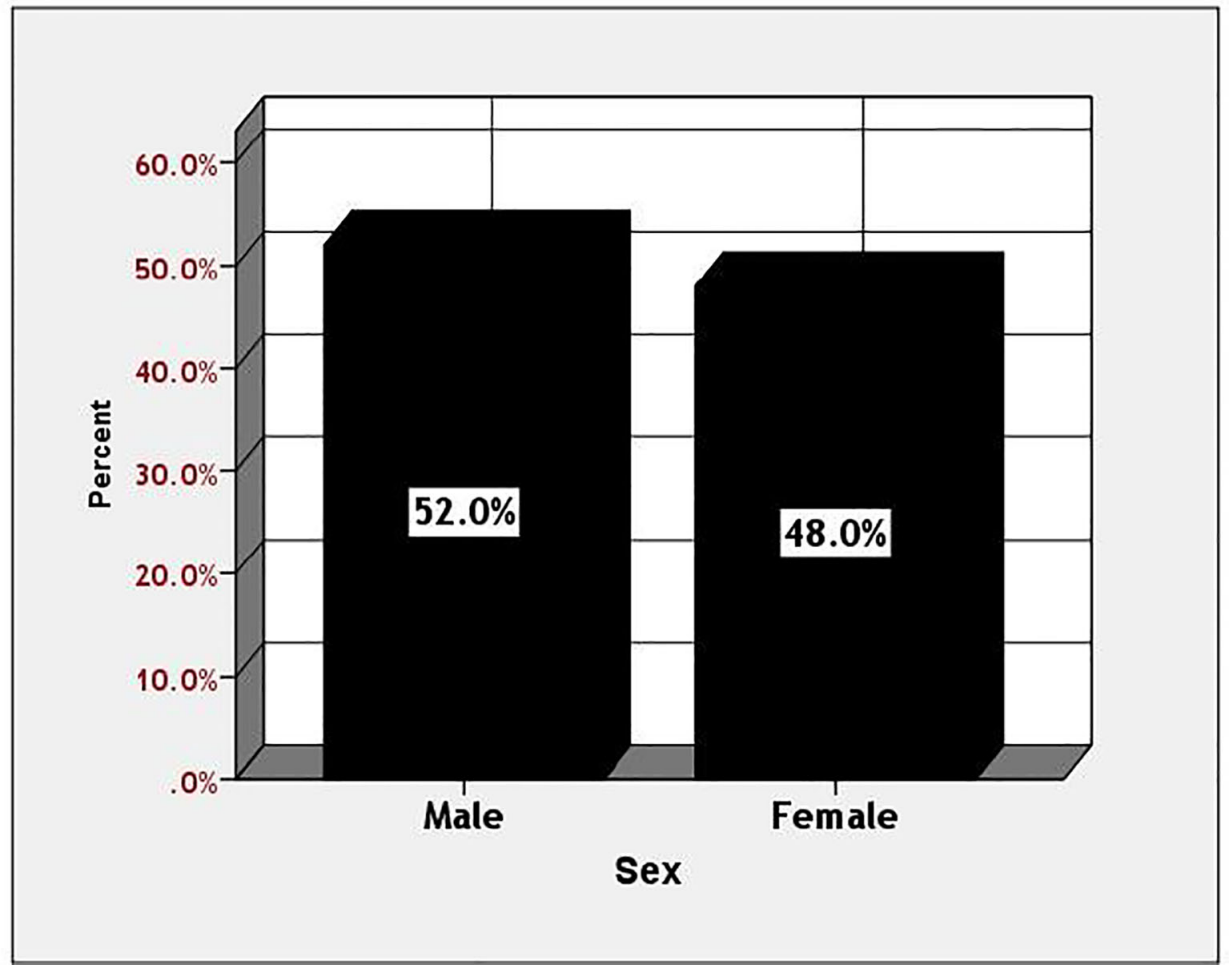
Prevalence of the studied diabetic patients according to the sex.

**Figure 2:**
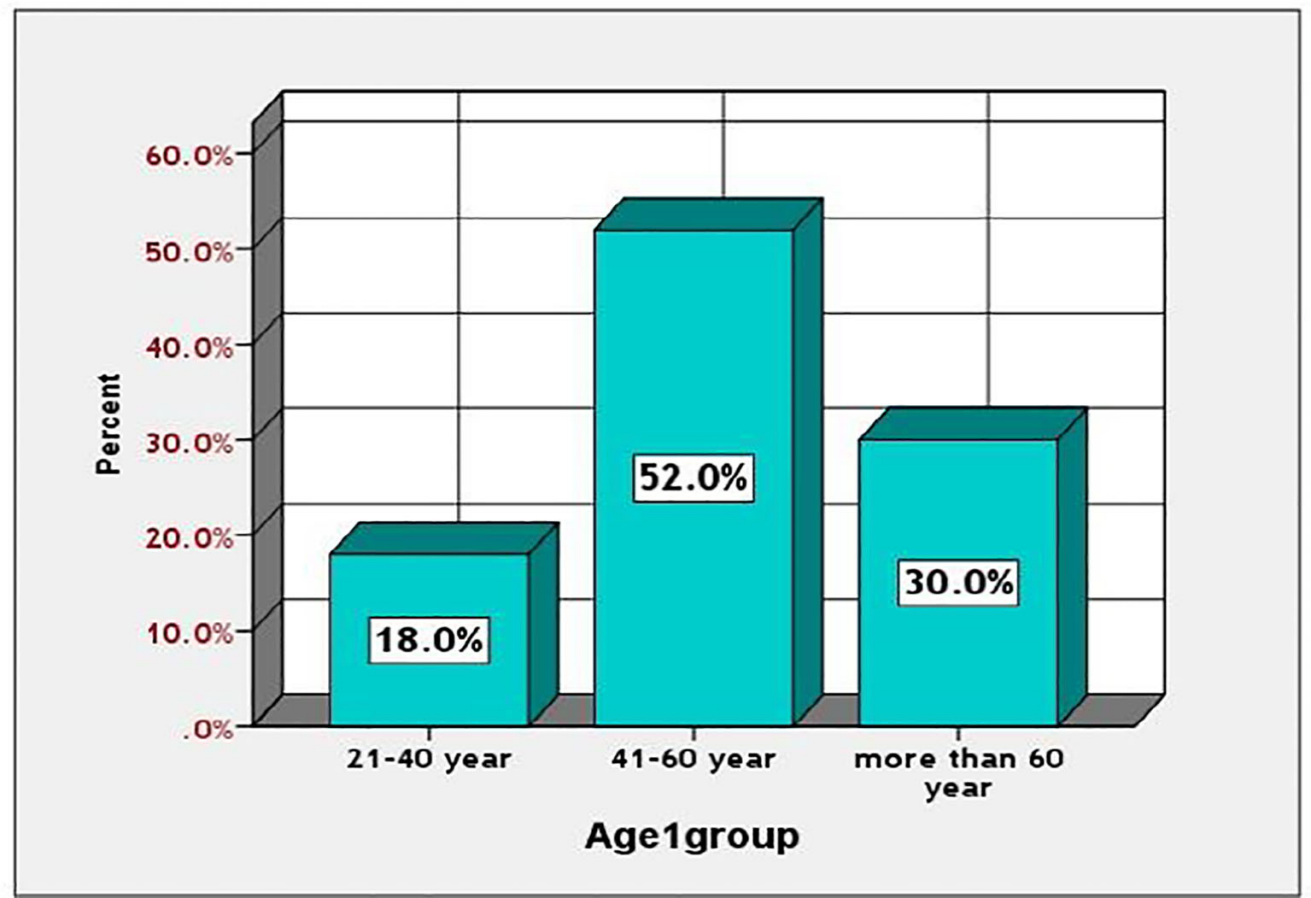
Prevalence of the studied diabetic patients according to the age.

Among diabetic patients, those aged 41 to 60 exhibited a significantly higher prevalence of diabetes mellitus (52%), compared to 18% in the 21 to 40 age group and 30% in individuals over 60. The prevalence was greater in men compared to women (52% versus 48%). This information is illustrated in **Figures 1**, **2**.

**Figure 3:**
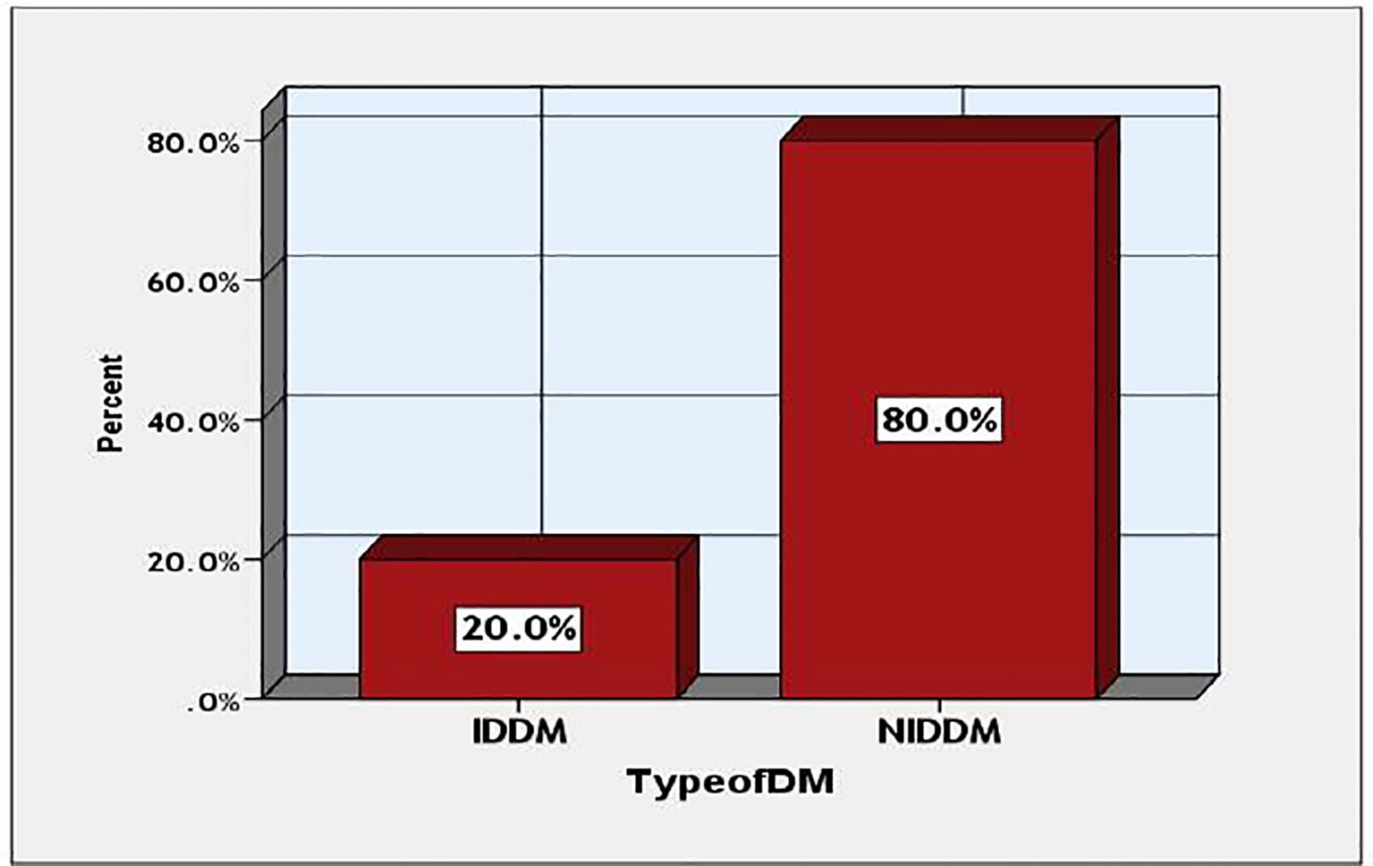
Prevalence types of DM among the studied participants.

This study examines the prevalence of various forms of diabetes among the participants. The data indicates that type 2 diabetes mellitus was significantly more prevalent than IDDM, with occurrences of 40 (80%) against 10 (20%), as illustrated in **Figure 3**.

**Figure 4:**
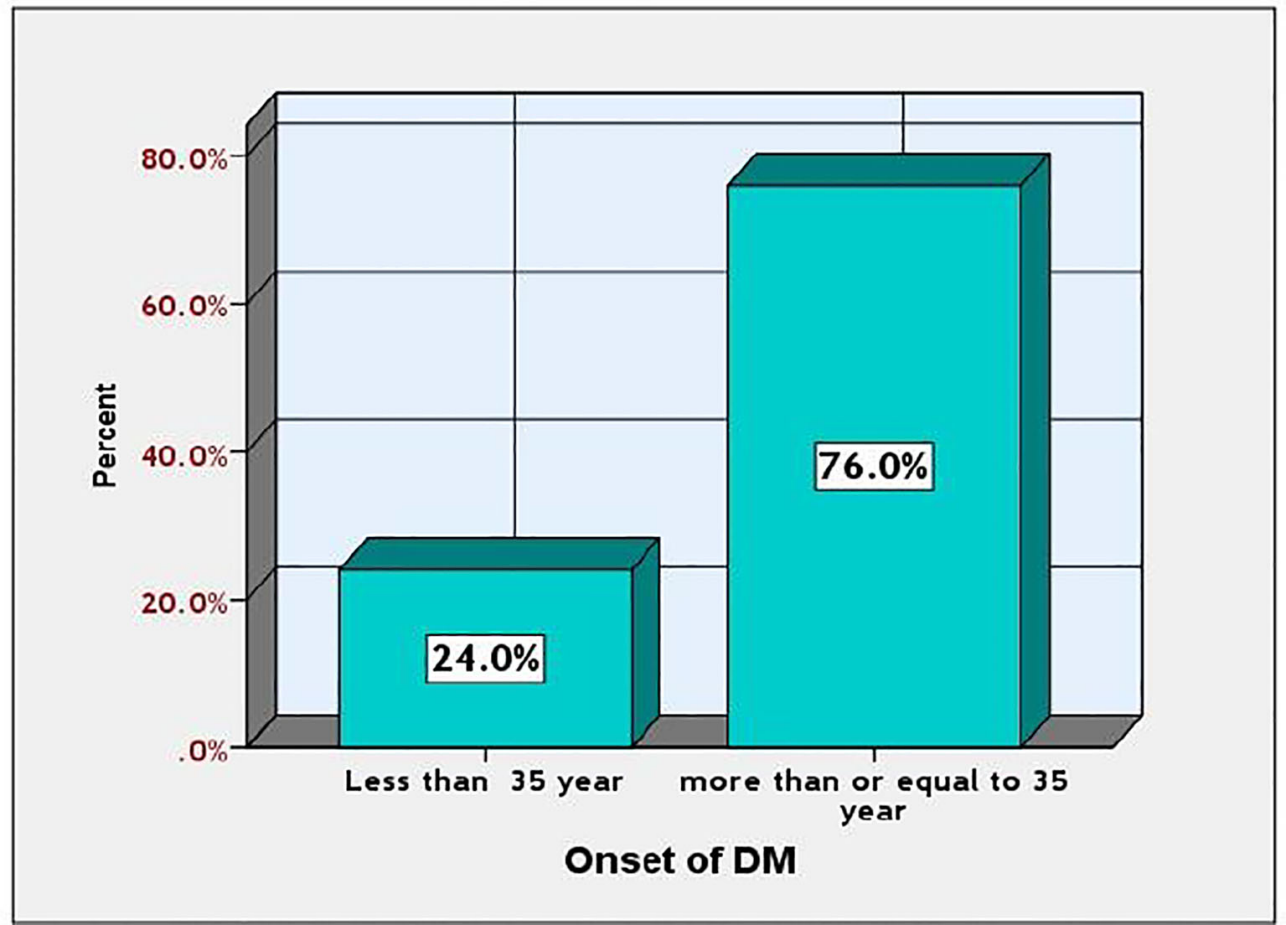
Prevalence onset of DM among the studied participants.

**Figure 4** illustrates the onset rate of diabetes mellitus (DM) among the studied diabetic patients. Diabetes mellitus (DM) prevalence was higher in individuals aged 35 and older compared to those younger than 35, with rates of 38.76% and 12.24%, respectively.

**Table 2** indicates that oral antidiabetic drugs were the most frequently utilized, accounting for 44%, followed by antihypertensives at 22%, insulin at 20%, and combination therapies at 14%. This corresponds with the elevated prevalence of type 2 diabetes mellitus and indicates a preference for pharmacological treatment rather than lifestyle modifications.

**Table 2:** Identify different type of the used DM drugs and their prevalence among the studied diabetic patients.

The used DM drugs	N	%
Insulin	10	20.0
Antihypertension	11	22.0
Oral anti diabetic agents	22	44.0
More than one drug	7	14.0

**Table 3** indicates that lab monitoring was significantly more prevalent in males (88.4%) compared to females (62.5%, p = 0.040). Other strategies, including exercise, treatment regulation, and nutrition therapy, exhibited no significant gender differences, suggesting possible disparities in access to diagnostic services.

**Table 3:** Distribution different type of management strategies of blood glucose level control among the studied diabetic patients according to the sex.

Different type of management strategies of blood glucose monitor		Male		Female		P value
		N	%	N	%	
Treatment regulation	Yes	11	42.3	13	54.2	0.403
	No	15	57.7	11	45.8	
Lab monitor	Yes	23	88.4	15	62.5	0.040
	No	3	11.6	9	37.5	
Exercise	Yes	6	23	5	20.8	0.848
	No	20	77	19	79.2	
Nutrition therapy	Yes	5	19.2	2	8.4	0.286
	No	21	80.8	22	91.6	

**Table 4** indicates a significant complication rate of 68%, with renal disease at 34% as the most prevalent, followed by retinopathy and neuropathy, each at 10%. This tables underscores the necessity for enhanced glycaemic regulation and prompt identification of complications.

**Table 4:** Identify complications of DM and their prevalence among the studied diabetic patients.

Complications of DM		N	%
Complications	Renal disease	17	34.0
	Retinal disease	5	10.0
	Gangrene on foot	2	4.0
	Cardiovascular disease	1	2.0
	Neuropathy	5	10.0
	More than one complication	4	8
No complications		16	32.0

**Table 5** indicates that BMI was significantly elevated in type 2 diabetic patients (28.2 vs. 20.0, p = <0.001). Other markers, including LDL, HDL, HbA1c, and FBS, were elevated in both groups; however, no statistically significant differences were observed. The findings underscore the significance of obesity in the context of type 2 diabetes.

**Table 5:** Mean of The clinical and biochemical blood level markers among the studied diabetic patients. .

The clinical and chemical blood level markers	IDDM	NIDDM	P value
	Mean ± SD	Mean ± SD	
LDL	128.3 ± 42.7	124.9 ± 43.6	0.823
HDL	41.0 ± 6.05	39.0 ± 0. 3	0. 253
TG	209.2 ± 58.7	245.7 ± 0.4	0.350
T Cholesterol	218.3 ± 43.9	201.6 ± 0.4	0.374
Creatinine	0.7 ± 0.3	0.71 ± 0.4	0.849
HbA1c	9.9 ± 0.3	9.5 ± 0.6	0.642
FBS	208.5 ± 81.9	179.8 ± 0.3	0.255
Dystolic BP	131.8 ± 30.4	132.0 ± 0.9	0.981
Cystolic BP	84.3 ± 19.0	86.8 ± 0.7	0.698
BMI	20.0 ± 2.4	28.2 ± 3.1	0.000

**Table 6** indicated that the prevalence of positive urine ketone bodies was significantly higher in IDDM patients (30% compared to 2.5%, p = <0.001, OR = 16.714), thereby identifying IDDM as a risk factor for ketosis. Additional markers such as proteinuria and glycosuria were more common in type 2 diabetes mellitus; however, they did not reach statistical significance.

**Table 6:** Results of qualitative tests of blood glucose level control among the studied diabetic patients.

Qualitative tests of blood glucose monitor		IDDM		NIDDM		95 CI		OR	P value
		N	%	N	%	Low	High		
Urine ketone body level	Positive	3	30	1	2.5	0. 080	7.518	16.714	0.000
	Negative	7	70	39	97.5				
Urine protein level	Positive	1	10	13	32.5	0. 495	37.928	0.230	0.185
	Negative	9	90	27	67.5				
Urine glucose level	Positive	6	60	15	37.5	0.097	1.651	2.500	0.205
Negative	4	40	25	62.5					

**Table 7** indicates that patients adhering to treatment exhibited significantly lower systolic blood pressure (83.2 vs. 89.0 mmHg, p = 0.022) and diastolic blood pressure (129.9 vs. 133.8 mmHg, p = 0.032).

**Table 7:** Effect of treatment regulation on the clinical and biochemical blood level markers among the studied diabetic patients.

The clinical and biochemical markers	Treatment Regulation Monitor				P value
	Yes		No		
	Mean	SD	Mean	SD	
LDL	119.6	36.6	131.0	48.0	0.363
HDL	39.8	5.6	39	3.8	0.802
TG	207.7	86.6	266.6	121.1	0.075
TCholesterol	190.7	42.7	217.9	58.2	0.061
Creatinine	0.7	0.3	0.6	0.2	0.028
HbA1c	8.5	2.0	10.4	2.3	0.114
FBS	180.4	70.8	190.3	71.4	0.149
DystolicBP	129.9	24.0	133.8	27.3	0.032
CystolicBP	83.2	16.0	89.0	19.1	0.022
BMI	26.3	4.6	26.7	6.1	0.603

**Table 8** presents lab monitoring results associated with lower triglyceride levels (220.9 vs. 293.5, p = 0.050) and HbA1c (9.2 vs. 10.4%, p = 0.002), indicating improved glycaemic control.

**Table 8:** Effect of lab monitor on the clinical and biochemical blood level markers among the studied diabetic patients.

The clinical and biochemical markers	Lab Monitor				P value
	Yes		No		
	Mean	SD	Mean	SD	
LDL	122.3	42.7	135.7	43.7	0.350
HDL	39.5	5.0	39.1	4.0	0.544
TG	220.9	101.0	293.5	119.3	0.050
TCholesterol	196.7	50.9	230.8	51.7	0.065
Creatinine	0.7	0.3	0.6	0.1	0.661
HbA1c	9.2	2.3	10.4	2.2	0.002
FBS	175.0	59.2	218.9	93.8	0.626
DystolicBP	134.9	28.3	122.6	10.6	0.596
Systolic	88.5	19.4	79.2	7.9	0.248
BMI	26.7	5.3	25.8	5.6	0.828

**Table 9** presents nutrition therapy. The intervention led to a significant reduction in triglycerides (164 vs. 250.4, p = 0.001), cholesterol levels (176.1 vs. 209.5, p = 0.023), and fasting blood sugar (121.1 vs. 196.0, p = <0.001).

**Table 9:** Effect of nutrition therapy on the clinical and biochemical blood level markers among the studied diabetic patients. .

The clinical and biochemical markers	Nutrition therapy				P value
	Yes		No		
	Mean	SD	Mean	SD	
LDL	107.0	31.2	128.5	44.0	0.143
HDL	42.1	6.2	39.0	4.4	0.239
TG	164.0	40.8	250.4	112.0	0.001
TCholesterol	176.1	27.1	209.5	54.5	0.023
Creatinine	0.7	0.3	0.6	0.1	0.471
HbA1c	7.8	2.5	9.8	2.2	0.098
FBS	121.1	34.6	196.0	69.6	0.000
Diastolic	122.8	9.5	133.4	27.1	0.064
Systolic	82.8	12.5	86.8	18.5	0.486
BMI	23.8	3.1	26.9	5.5	0.057

**Table 10** indicates that educated patients exhibited a significantly lower BMI (25.1 vs. 28.7, p = 0.025) and generally superior biochemical profiles, although the majority of other differences did not reach statistical significance. This demonstrates the beneficial impact of health literacy on diabetes management.

**Table 10:** The association between the clinical and biochemical blood level markers and educational level among the studied diabetic patients.

The clinical and biochemical markers	Educational Level				P value
	Literacy		Education		
	Mean	SD	Mean	SD	
LDL	128.7	48.0	123.4	39.8	0.683
HDL	38.3	4.7	40.1	4.7	0.193
TG	260.0	129.5	223.9	92.4	0.290
T.Cholesterol	206.7	53.7	202.2	52.3	0.634
Creatinine	0.7	0.4	0.6	0.1	0.459
HbA1c	9.8	2.3	9.3	2.4	0.473
FBS	190.7	55.2	182.1	79.9	0.653
Diastolic	136.2	23.6	129.1	26.9	0.330
Systolic	87.7	14.4	85.3	19.8	0.629
BMI	28.7	5.7	25.1	4.7	0.025

**Table 11** indicates that females exhibited a higher BMI (28.0 vs. 25.1, p = 0.058) and elevated levels of most biomarkers, although these differences did not reach statistical significance. These trends may suggest distinct risk patterns or variations in care based on gender.

**Table 11:** The association between the clinical, biochemical blood level markers and the gender among the studied diabetic patients.

The clinical and biochemical markers	Gender				P value
	Male		Female		
	Mean	SD	Mean	SD	
LDL	122.5	37	128.8	49.1	0.616
HDL	39.9	5.1	39.0	4.5	0.497
TG	236.8	103.2	240.0	117.2	0.918
TCholesterol	200.2	50.0	209.9	56.1	0.522
Creatinine	0.8	0.3	0.6	0.2	0.132
HbA1c	9.3	2.4	9.7	2.3	0.612
FBS	175.0	62.5	196.9	78.2	0.284
Diastolic BP	128.3	26	135.9	25.3	0.302
Systolic	83.8	19.5	89.0	15.7	0.300
BMI	25.1	4.3	28.0	6.0	0.058

**Table 12** showed that non-adherence to management methods (treatment, exercise, and diet) was related to a higher incidence of problems; however, this was not statistically significant. Exercise and nutrition had odds ratios of 1.333 and 1.207, respectively, indicating a potential risk of problems in the absence of these interventions.

**Table 12:** The association between different type of management strategies of blood glucose level control and complications of DM among the studied diabetic patients.

Type of management strategies of blood glucose monitor		Complications				OR	P value
		Yes		No			
		N	%	N	%		
Treatment regulation monitor	Yes	14	41.2	10	62.5	0.420	0.164
	No	20	58.8	6	37.5		
Lab monitor	Yes	24	70.6	14	87.5	0.343	0.205
	No	10	29.4	2	12.5		
Exercise	Yes	8	23.5	3	18.8	1.333	0.709
	No	26	76.5	13	81.2		
Nutrition therapy management	Yes	5	14.7	2	12.5	1.207	0.834
	No	29	85.3	14	87.5		

## Discussions

The investigation revealed that kidney disorders were the most common complication in individuals with diabetes, followed by eye disorders, nerve damage, foot gangrene, and heart-related conditions. This is consistent with the findings of Mazin Yousif Elhendi (18), who similarly identified these as prevalent complications among diabetic patients. Changes in metabolism and blood flow associated with diabetes lead to glomerular sclerosis and fibrosis, which exacerbate diabetic nephropathy.

The study indicated that non-treatment regulation (52%) and lack of nutritional therapy (86%) were more common than their respective alternatives. Laboratory monitoring was the predominant strategy, utilized by 76% of participants. These findings align with those of Ahmed Ismail Albarrak (19), who observed a low compliance rate with ADA standards of care at 36.3%. However, documentation of elements such as medical history, meal planning, and glucose monitoring occurred more frequently. Mazin Yousif Elhendi (18) reported comparable trends, indicating treatment regulation at 92.7%, laboratory monitoring at 74%, and dietary management at 26.8%.

Patients adhering to treatment regulations exhibited reduced levels of LDL, triglycerides (TG), total cholesterol, HbA1c, fasting blood sugar (FBS), and BMI, alongside significantly lower blood pressure. Interestingly, these patients showed elevated creatinine levels, possibly due to the administration of newer medications like GLP-1 receptor agonists, which enhance insulin secretion and reduce postprandial glucose fluctuations. These findings are supported by both Mazin Yousif Elhendi (18) and Diabetes UK (2010) (20), emphasizing the importance of effective therapy in mitigating diabetes-related complications.

Individuals adhering to a dietary regimen exhibited reduced levels of LDL, HbA1c, blood pressure, and BMI, along with significantly decreased TG, total cholesterol, and FBS. These results are consistent with recommendations from the American Diabetes Association (ADA) (21) which has highlighted the effectiveness of nutrition therapy in lowering blood glucose, lipid levels, body weight, and blood pressure. Additionally, Hawthorne (22) emphasized the role of dietary guidance in managing weight, hypertension, and glycaemic control among diabetic patients.

Patients who engaged in regular physical activity showed significantly decreased levels of LDL, BMI, TG, total cholesterol, creatinine, HbA1c, and FBS. These findings are in line with those of the DCCT Research Group (23) which stressed the importance of exercise in maintaining normoglycemia and preventing long-term diabetes complications.

Diabetic patients who received education exhibited reduced levels of LDL, TG, total cholesterol, creatinine, HbA1c, FBS, and blood pressure, along with significantly lower BMI. Diabetes self-management education (DSME) plays a critical role in preventing or delaying complications associated with diabetes. Studies by (24, 25) demonstrated that DSME improves glycaemic control. Further support comes from Marden (26) and Chaudhary (27), who emphasized the importance of literacy and education in enhancing outcomes and reducing complications.

Non-adherence to treatment, exercise, or nutrition therapy was associated with an increased prevalence of complications among patients. In contrast, individuals who underwent regular laboratory monitoring experienced a reduced incidence of complications. These results are supported by the American Diabetes Association (2002) (21), Diabetes UK (2010) (20), and the DCCT Research Group (1995) (23) all of whom emphasize the importance of strict metabolic control in preventing diabetes complications.

### Study limitations

This study has several limitations. Firstly, the relatively small sample size of 50 diabetic patients limits the statistical power of the analysis and reduces the generalizability of the results to the wider diabetic population in Sana’a city and Yemen. Secondly, the recruitment was limited to hospitalized patients present during the researchers’ visits, which may introduce selection bias by overrepresenting patients with more severe disease or complications. This sampling method limits the applicability of findings to community-dwelling or outpatient diabetic populations, who may have different management experiences and outcomes. Additionally, the cross-sectional design provides only a snapshot of diabetes management and does not allow for assessment of causal relationships or changes over time. Self-reported data on treatment adherence, exercise, and nutrition may be subject to recall or social desirability bias, potentially affecting the accuracy of reported behaviors. Finally, some biochemical and clinical parameters were measured only once, which may not fully capture fluctuations in blood glucose control or other metabolic variables.

Our study suggests several recommendations to improve diabetes management in hospitals across Sana’a:

Enhance patient education, especially for those with low literacy levels, strengthen adherence support for treatment, nutrition, and exercise, increase regular laboratory monitoring, particularly for women, establish multidisciplinary care teams, expand outpatient and community-based follow-up, Improve healthcare resources and infrastructure. these measures could significantly improve patient outcomes and reduce the incidence of diabetes-related complications.

## Conclusions

This study highlights that diabetes mellitus in Sana’a city predominantly affects middle-aged, married, and unemployed individuals with low educational levels. Non-insulin-dependent diabetes mellitus is the most common form, and renal complications are prevalent among patients. Laboratory monitoring is the most commonly employed management strategy, while adherence to treatment, exercise, and nutrition remains suboptimal, especially among females. Patients who adhered to treatment, nutritional guidance, and laboratory monitoring exhibited better biochemical profiles and reduced complication rates, underscoring the importance of comprehensive diabetes management. Education and gender differences influence disease control and outcomes, suggesting the need for targeted interventions.

Given the study’s limitations, including a small, hospital-based sample, further research with larger and more representative populations is necessary. Strengthening patient education, improving lifestyle adherence, and enhancing access to regular monitoring are critical steps to reduce the burden of diabetes complications in this population.

## Data Availability

The original contributions presented in the study are included in the article/supplementary material. Further inquiries can be directed to the corresponding author.
